# Unhealthy Lifestyle Is an Important Risk Factor of Idiopathic BPPV

**DOI:** 10.3389/fneur.2020.00950

**Published:** 2020-10-15

**Authors:** Chang-yong Fu, Zhen-zhong Zhang, Jin Chen, Sandip Kumar Jaiswal, Fu-ling Yan

**Affiliations:** ^1^School of Medicine, Southeast University, Nanjing, China; ^2^Neurology Department, Tongde Hospital of Zhejiang Province, Hangzhou, China; ^3^Department of Neurology, Zhongda Hospital, Southeast University, Nanjing, China

**Keywords:** BPPV-benign paroxysmal positional vertigo, risk factors, physical activity, quarantine policy, recumbent position time

## Abstract

**Background:** Benign paroxysmal positional vertigo (BPPV) is a self-limiting and recurrent disease but the cost is considerable. The number of patients with BPPV increased significantly under the quarantine policy in Hangzhou. The unhealthy lifestyle risk factors of BPPV have not yet been investigated. Thus, the objective is to analyze whether an unhealthy lifestyle is a risk factor of BPPV.

**Methods:** One hundred and sixty three patients with idiopathic BPPV aged 22–87 years (BPPV group), and 89 aged 23–92 years sex-matched control subjects (non-BPPV group) were enrolled in this study. All BPPV patients received a definitive diagnosis which excluded secondary BPPV. Non-BPPV cases excluded BPPV, sudden deafness, Meniere's disease, ear or craniofacial surgery, vestibular neuritis, and head trauma history. We obtained a blood lipids profile, serum uric acid, total bilirubin, and related diagnostic information through the electronic medical record system. To get the time of physical activities and recumbent positions, we asked the patient or their family from February 2020 to June 2020, and the rest of the patient's information was acquired by phone or WeChat.

**Data Analyses:** The *t*-test or chi-squared test, univariate, and multiple logistic regression analyses were performed for the two groups. For each factor, odds ratios were calculated with 95% confidence intervals (CIs). Moreover, test equality of two or more receiver operating characteristic (ROC) analyses were applied to the physical activities, and recumbent position time; area under curve (AUC) measures were calculated with 95% CIs and compared with each other.

**Results:** The BPPV group had unhealthy lifestyles such as poor physical activities, prolonged recumbent position time, and low rate of calcium or VD supplementation in univariate logistic regression analyses (*P* < 0.05). Poor physical activities and prolonged recumbent position time were independently associated with BPPV in multiple logistic regression models (OR = 18.92, 95% CI: 6.34–56.43, *p* = 0.00 and OR = 1.15, 95% CI: 1.01–1.33, *p* < 0.04). In the comparison of ROC curves of recumbent position time and physical activities in identifying BPPV, AUCs were 0.68 (0.61–0.74), and 0.68 (0.63–0.73), respectively.

**Conclusion:** We conclude that poor physical activities and prolonged recumbent position time may be independent risk factors for BPPV patients, but hypertension, hyperuricemia, hyperlipidemia, hemoglobin, diabetes, serum bilirubin, CHD, and CI, may not be.

## Background

Vertigo is one of the most common symptoms in neurological illness and the cost of evaluating dizziness is considerable. Benign paroxysmal positional vertigo (BPPV) is the most common peripheral vertigo disease (1). It amounts to 20% of all vertigo patients (2). BPPV has a recurrence rate of about 15% every year (3). Some studies have found that the age, gender, hypertension, hyperuricemia, hyperlipidemia, diabetes, and osteoporosis may be the risk factors of BPPV (4, 5). There are few studies on the unhealthy lifestyle and BPPV.

**Figure 1:**
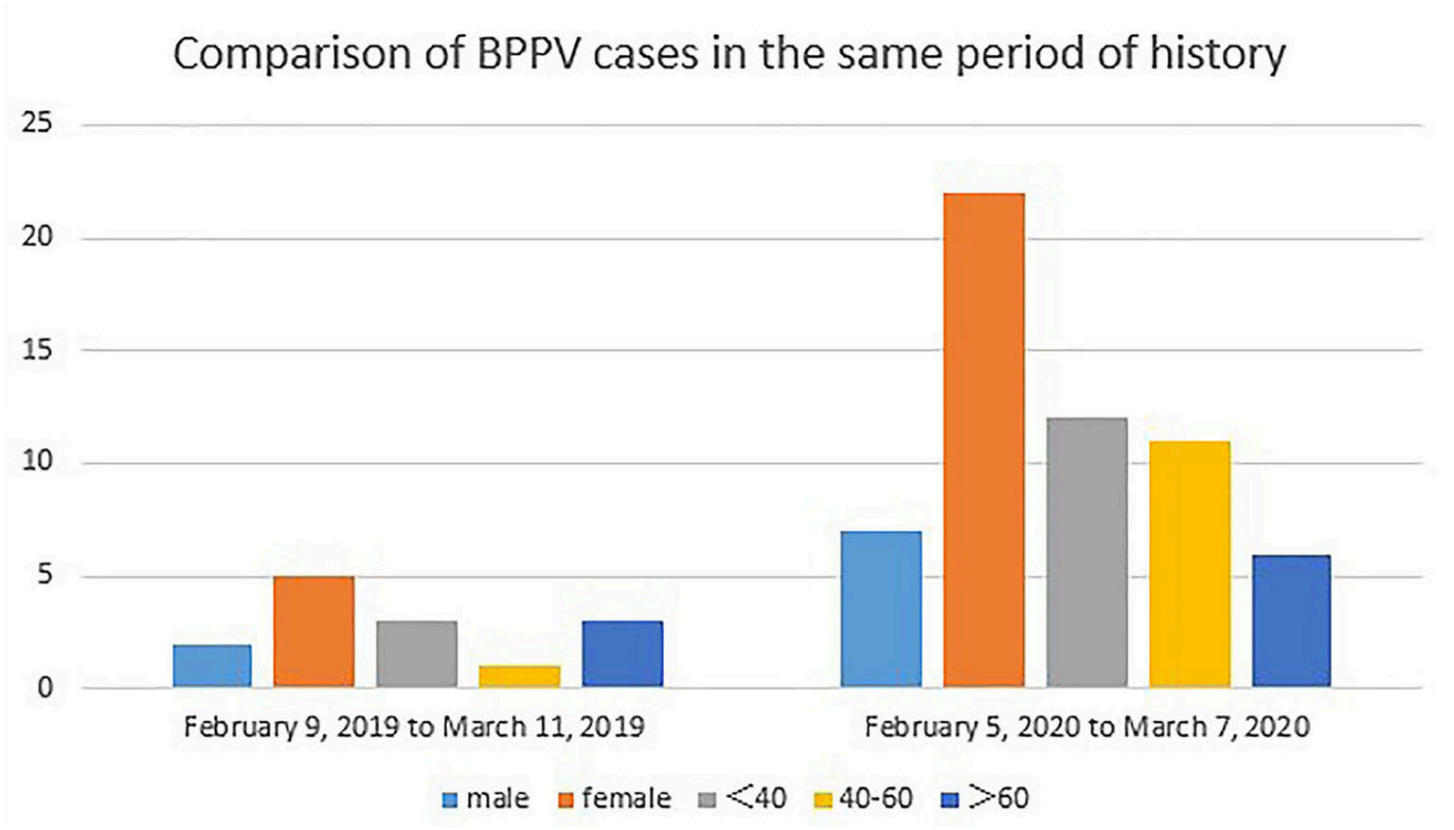
The BPPV of young and middle-aged and total cases increased significantly during the period of 30 days in the quarantine policy of COVID-19 than in the same period last year.

After the quarantine policy was performed to prevent COVID-19 in Hangzhou, it was found that the number of BPPV diagnoses increased more rapidly than in the same period in 2019 (Figure 1). Therefore, in this study, we aimed to (1) investigate the risk factors of BPPV; and (2) explore the association between an unhealthy lifestyle and BPPV. We hypothesized that the onset of BPPV is associated with people's unhealthy lifestyles.

## Methods

### Object of Study

A retrospective observational study was conducted in the Department of Neurology in Tongde Hospital of Zhejiang Province from June 16, 2018 to June 30, 2020. The study included 163 patients with idiopathic BPPV aged 22–87 years (BPPV group), and 89 aged 23–92 years sex-matched control subjects (non-BPPV group). The BPPV group patients received a definitive diagnosis and CRM treatment, and excluded secondary BPPV. The non-BPPV group enrolled patients who after an annual physical examination in our hospital excluded diagnoses for BPPV, sudden deafness, Meniere's disease, ear or craniofacial surgery, vestibular neuritis, and head trauma. We obtained a blood lipids profile, serum uric acid, total bilirubin, and related diagnostic information through the electronic medical record system. To get the time of physical activities and recumbent position we asked the patients or their family from February 2020 to June 2020, and the rest of the patient's information was acquired by phone or WeChat.

### Diagnostic Criteria

This references the diagnosis standard of BPPV in the 2014 New England Journal (6). All the patients were examined by Dix-Hallpike and roll-tested.

The definition of hyperuricemia is based on the laboratory standard of our hospital that states that serum uric acid in female patients must be higher than 340 μmol/L and in male patients higher than 400 μmol/L. Hyperlipidemia is defined when low-density lipoprotein is higher than 3.2 mmol/L, total cholesterol is higher than 5.7 mmol/L, or triglyceride is higher than 1.95 mmol/L. Lack of physical activity is defined as <5 exercises per week and <20 min at a time. The prolonged recumbent position time is defined as when the daily lying time is longer than or equal to 10 h, including the time of falling asleep and not falling asleep.

If the patient had suffered from the following diseases, it will be classified as secondary benign paroxysmal positional vertigo and will be excluded. Such as sudden deafness, Meniere's disease, ear or craniofacial surgery, vestibular neuritis within 1 year, or head trauma within 1 year.

### Data Analyses

The measurement data in accordance with normal distribution are expressed by $\bar{x}$ ± S, and the comparison between groups is expressed by the *t*-test; the numeration data were statistically analyzed with the chi-squared test. When *p* < 0.05, the differences between the two groups were deemed to be statistically significant (Table 1). Multivariable logistic regression was performed to identify the risk factors of BPPV in all of the patients (Table 2). The comparison of ROC curves of recumbent position time and physical activities are shown in (Figure 1). For each factor, odds ratios were calculated with 95% confidence intervals (CIs). All statistical analyses were performed using the STATA statistical software version 15.1.

**Table 1:** Univariate analysis of BPPV related risk factors (*N* = 252).

**Variables**	**BPPV group (*****n*** **=** **163)**		**Non-BPPV group (*****n*** **=** **89)**		**Odds ratio**	**95% CI**	***p*-value**
	**Total (*N*)**	**Column (%)**	**Total (*N*)**	**Column (%)**			
Age, years (mean ± SD)	56.46 ± 15.67		54.47 ± 18.37			53.69–57.82	0.37
Female	105	64.42	60	57.42	0.88	0.49–1.56	0.63
Hypertension	46	28.22	29	32.58	0.81	0.45–1.49	0.47
Low-density lipoprotein	2.73 ± 0.80		2.79 ± 0.76			2.65–2.85	0.58
>3.2 mmol/L	43	26.38	23	25.84	1.03	0.55–1.95	0.93
Total cholesterol	4.50 ± 1.09		4.54 ± 1.08			4.38–4.65	0.78
>5.7 mmol/L	21	12.88	12	13.48	0.95	0.42–2.25	0.91
Triglyceride	1.59 ± 1.43		1.40 ± 0.79			1.37–1.68	0.25
>1.95 mmol/L	37	22.70	17	19.10	1.24	0.63–2.53	0.51
Hemoglobin	131.21 ± 17.67		132.58 ± 18.39			129.47–133.92	0.56
Diabetes	14	8.59	8	8.99	0.95	0.35–2.73	0.91
Osteoporosis	3	1.84	4	4.49	0.40	0.06–2.42	0.22
Serum uric acid	311.46 ± 87.10		307.67 ± 91.44			299.14–321.10	0.745
Male > 400 μmol/L.	10	6.13	4	4.49	1.39	0.39–6.24	0.59
Female > 340 μmol/L	39	23.93	20	22.47	1.09	0.57–2.13	0.79
Serum bilirubin	13.80 ± 6.14		14.87 ± 6.55			13.40–14.96	0.20
Vitamin D supplement	3	1.84	7	7.87	0.22	0.04–1.00	0.02*
Calcium supplement	3	1.84	8	8.99	0.19	0.03–0.82	0.01*
Fracture of history	4	2.45	4	4.49	0.53	0.97–2.95	0.37
CHD	10	6.13	9	10.11	0.58	0.20–1.69	0.25
CI	13	7.98	9	10.11	0.77	0.29–2.14	0.57
Poorphysical activities	159	97.55	55	61.80	24.57	8.12–98.22	0.00*
Recumbent position Time (mean ± SD)	10.37 ± 2.78		8.98 ± 2.36			9.54–10.22	0.0001*
≥10 h	96	58.90	28	31.46	3.12	1.75–5.61	0.00*

* *Prolonged recumbent position time, poor physical activities, Vitamin D and Calcium supplement reached statistical significance (P < 0.05). SD, standard deviation; CHD, coronary heart disease; CI, cerebral infarction*.

**Table 2:** Multiple logistic regression analysis to identify the predictors of risk factors for BPPV.

**Variables**	**Odds ratio**	**95% confidence interval**	***p*-value**
Age	1.01	0.99–1.03	0.43
Low-density lipoprotein	0.82	0.46–1.46	0.51
Total cholesterol	0.93	0.59–1.47	0.75
Triglyceride	1.27	0.87–1.87	0.22
Serum uric acid	1.00	0.99–1.00	0.78
Calcium supplement	0.11	0.01–1.38	0.09
Vitamin D supplement	0.66	0.04–10.38	0.77
Poor physical activities	18.92	6.34–56.43	0.00*
Recumbent position Time (mean ± SD)	1.15	1.01–1.33	0.04*

* *Poor physical activities and prolonged recumbent position time were significance predictors of BPPV*.

## Results

Univariate analysis of BPPV related risk factors of the two group's patients were summarized in Table 1. No significant difference was found between the two groups with respect to age, MD, sudden deafness migraine, hypertension, hyperlipidemia, CHD, CI, and diabetes (*P* > 0.05). Although lifestyles including prolonged recumbent position time (≥10 h) (OR = 3.12, 95% CI: 1.75–5.61, *P* = 0.00), and poor physical activities (OR = 24.57, 95% CI: 8.12–98.22, *P* = 0.00) reached statistical significance in patients with BPPV compared with controls.

To identify the predictors of BPPV, multiple logistic regression analyses were performed. Due to the strong correlation, recumbent position time and poor physical activities values were found to be independently associated with BPPV in the multiple logistic regression model (Table 2). Multivariable logistic regression revealed that prolonged recumbent position time (OR = 1.15, 95% CI: 1.01–1.33, *p* < 0.04), and poor physical activities (OR = 18.92, 95% CI: 6.34–56.43, *p* = 0.00) may be important risk factors for BPPV (Table 2).

**Figure 2:**
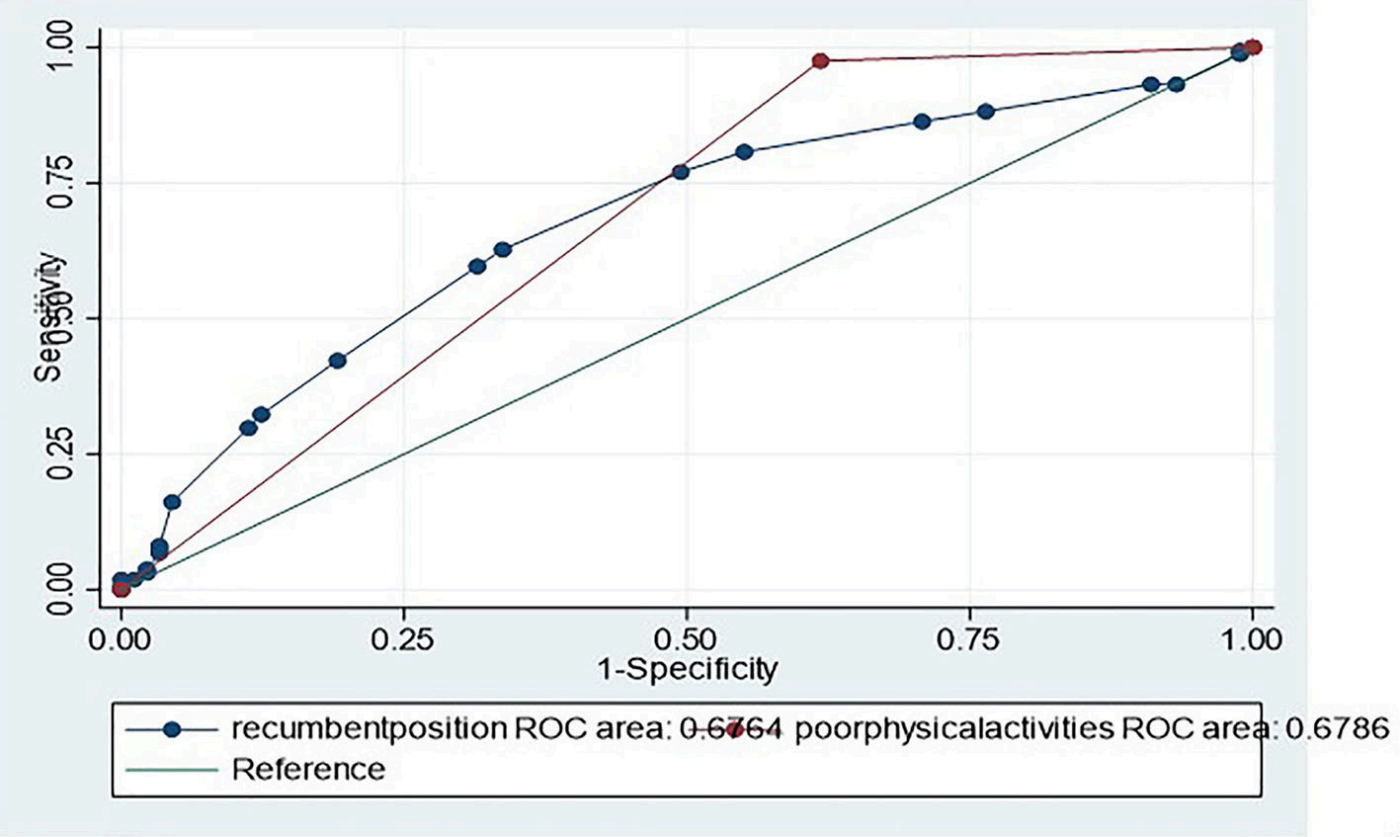
Comparison of ROC curves of recumbent position time and poor physical activities in identifying BPPV. AUCs were 0.68 (0.61–0.74), and 0.68 (0.63–0.73), respectively.

Receiver operating characteristic analyses were applied to recumbent position time and physical activities variables. AUCs were 0.68 (0.61–0.74), and 0.68 (0.63–0.73), respectively (Figure 2).

## Discussion

As we all know, age, gender, sex hormones, osteoporosis, hypertension, hyperlipidemia, diabetes, plasma vitamin D level, and hyperuricemia are all considered as risk factors for BPPV (4, 5, 7–10). According to a previous study, cerebrovascular risk factors influence BPPV onset (9, 11). In addition, some studies have found that age does not increase the recurrence rate of BPPV (11) and seasonal vitamin D deficiency in winter is not enough to cause BPPV (12). The risk factor of BPPV needs further analysis. In theory, with the increase of age, the function of human organs gradually declines and cardiovascular risk factors increase with age. As a part of the inner ear structure, the metabolism, absorption, and regeneration of otoliths are affected, and can easily fall off and lead to BPPV. Previous studies have found that the high morbidity of BPPV in women may be related to widespread osteoporosis (6). It may also be related to the abnormal hormone metabolism in post-menopausal women. We found that there was no obvious correlation to the common BPPV related risk factors in this study, such as hypertension, hyperuricemia, hyperlipidemia, diabetes, serum bilirubin, CHD, and CI.

We found that the numbers of idiopathic BPPV was significantly higher than the same period a year earlier under the quarantine policy in Hangzhou from January 2020 to March 2020. This may correlate with the unhealthy lifestyle of patients during the COVID-19 spread. To verify this hypothesis, we expanded the sample size of idiopathic BPPV and set up a non-BPPV health checker as a control group.

As to the lifestyle of the BPPV group, the majority of patients had the following characteristics, poor physical activities and prolonged recumbent position time. It can be seen that prolonged recumbent position time and poor physical activities may be important pathogenic factors for BPPV. Van WE confirmed that 11% of the dizziness symptoms in Parkinson's patients are likely to be BPPV, which is also considered to be related to poor physical activities (13). It has been suggested that a prolonged recumbent position may promote calcium carbonate deposition and otolith relaxation in the elliptical capsule (4). The author believes that this view can explain the mechanism of the significant increase of BPPV patients in our study. Studies have found that poor physical activities is one of the most important risk factors for BPPV in women and the morbidity of women who do not exercise is 2.62-fold that of women who regularly exercise (14). Regular physical exercise may be a good choice to prevent BPPV.

Some studies found that the decrease in the plasma vitamin D level is directly related to BPPV (9, 10). We believe that prolonged recumbent position time and poor physical activities can lead to sunlight insufficiency, which in turn leads to vitamin D deficiency.

Through this clinical study, we hypothesized that the broken otolith of the endolymph in healthy people may be continuous, which may be absorbed and dissipated due to regular exercise and suitable recumbent position time. For those who have prolonged recumbent position time or poor regular physical activities, the deposition is affected by gravity, and when they move position such as getting up from a resting position or turning over, it may result in BPPV. This finding may explain why BPPV occurs several days after trauma, rather than immediately after trauma.

The movement of the body and head may promote the circulation of the endolymph in the semicircular canal, and the degenerative otolith also dissolves and dissipates with the circulation. However, prolonged recumbent position time or poor regular physical activities will slow down the circulation. The otolith particles in the membranous labyrinth will also increase due to the unhealthy lifestyle. The three-dimensional movement of the body and head may promote the formation of the normal structure and functional remodeling of otoliths on the utricle. However, an unhealthy lifestyle may lead to otolith structural disorder, which may lead to the otolith falling off easily.

## Conclusion

This study found that idiopathic BPPV had no obvious relationship with hypertension, hyperuricemia, hyperlipidemia, hemoglobin, diabetes, serum bilirubin, CHD, or CI. In this study, poor physical activities and prolonged recumbent position time are important predictors for BPPV. Changing unhealthy lifestyles may be the solution to decrease the morbidity of BPPV. The authors speculate that BPPV is associated with poor physical activities and prolonged recumbent position time which may be the independent risk factors.

The limitations of the study are that it failed to assess the anxiety and depression of all patients. Sleep quality was not included in the analysis (15). BPPV styles were not classified. Osteoporosis information was obtained only through asking the patient for their medical history, lacking relevant examinations.

## Data Availability Statement

All datasets presented in this study are included in the article/Supplementary Material.

## Ethics Statement

The studies involving human participants were reviewed and approved by Ethics Committee of Tongde Hospital of Zhejiang Province. Written informed consent for participation was not required for this study in accordance with the national legislation and the institutional requirements. Written informed consent was obtained from the individual(s) for the publication of any potentially identifiable images or data included in this article.

## Author Contributions

C-yF conceived the study and design, conducted the experiment, and wrote the manuscript. F-lY prepared manuscript, and revised this manuscript. Z-zZ, JC, and SJ conducted the acquisition of subjects and the interpretation of data. All authors contributed to the article and approved the submitted version.

## Conflict of Interest

The authors declare that the research was conducted in the absence of any commercial or financial relationships that could be construed as a potential conflict of interest.

## Abbreviations

BPPV: Benign paroxysmal positional vertigo
SD: standard deviation
CHD: coronary heart disease
CI: cerebral infarction
AUC: area under curve
ROC: receiver operating characteristic
MD: Meniere's disease.
